# Na/K-ATPase Y260 Phosphorylation–mediated Src Regulation in Control of Aerobic Glycolysis and Tumor Growth

**DOI:** 10.1038/s41598-018-29995-2

**Published:** 2018-08-17

**Authors:** Moumita Banerjee, Xiaoyu Cui, Zhichuan Li, Hui Yu, Liquan Cai, Xuelian Jia, Daheng He, Chi Wang, Tianyan Gao, Zijian Xie

**Affiliations:** 1 Marshall Institute for Interdisciplinary Research (MIIR), Marshall University, Huntington, West Virginia 25703 USA; 2 Department of Physiology and Pharmacology and Medicine, University of Toledo College of Medicine, Toledo, Ohio, 43614 USA; 3 Department of Cancer Biostatistics, Markey Cancer Research Center, University of Kentucky, Lexington, Kentucky 40536 USA; 4 Department of Molecular and Cellular Biochemistry, Markey Cancer Research Center, University of Kentucky, Lexington, Kentucky 40536 USA; 5 Present Address: Department of Pediatrics, Union Hospital, Tongji Medical College, Huazhong University of Science and Technology, Wuhan, Hubei 430022 China

## Abstract

We report here the identification of α1 Na/K-ATPase as a major regulator of the proto-oncogene Src kinase and the role of this regulation in control of Warburg effect and tumor growth. Specifically, we discovered Y260 in α1 Na/K-ATPase as a Src-specific phosphorylation and binding site and that Y260 phosphorylation is required for Src-mediated signal transduction in response to a number of stimuli including EGF. As such, it enables a dynamic control of aerobic glycolysis. However, such regulation appears to be lost or attenuated in human cancers as the expression of Na/K-ATPase α1 was significantly decreased in prostate, breast and kidney cancers, and further reduced in corresponding metastatic lesions in patient samples. Consistently, knockdown of α1 Na/K-ATPase led to a further increase in lactate production and the growth of tumor xenograft. These findings suggest that α1 Na/K-ATPase works as a tumor suppressor and that a loss of Na/K-ATPase-mediated Src regulation may lead to Warburg phenotype in cancer.

## Introduction

Cancer cells show increased dependence on aerobic glycolysis for energy utilization, a phenomenon known as Warburg effect. This metabolic switch provides important metabolites for cancer cells. The protooncogene Src kinase is known to drive Warburg effect in cancer cells by phosphorylating metabolic enzymes and is frequently hyperactivated in cancer^1–5^. Although a lot is known about the structural regulation of Src activity it is not sufficient to explain this apparent hyperactivation in cancer. Till now, a major plasma membrane regulator of Src has not been identified where Src participates in transmitting signal from multiple cell surface receptors^2,6^.

The Na/K-ATPase is a highly expressed membrane protein and the plasma membrane contains more than a million of them in most human cells. Interestingly, only about 30% of the plasma membrane Na/K-ATPase is engaged in ion pumping^7^. Recent studies have uncovered that the α1 Na/K-ATPase interacts with Src to form a receptor complex that allows endogenous cardiotonic steroids (CTS) to initiate protein and lipid kinase cascades through EGF receptor/reactive oxygen species (ROS) pathways, thereby regulating an array of cellular activities^8–10^. However, whether this interaction is important for the regulation of the plasma membrane pool of Src is unknown.

We have previously demonstrated that the α1 isoform of Na/K-ATPase interacts directly with Src kinase through two pairs of domain interactions^7,11,12^. While the second cytosolic domain of α1 subunit acts like a Src SH2 ligand involved in the activation and targeting of Src, the nucleotide binding domain of α1 binds the kinase domain and keeps Src in an inactive state^11–15^. Interestingly, although there are four isoforms of α subunit, only α1 appears to interact with Src kinase^16,17^.

In this study, we took the advantage of this isoform-specific Src regulation and identified Y260 in α1 Na/K-ATPase as a key to the formation of a protein complex in the plasma membrane that allows dynamic regulation of Src-mediated signal transduction in response to many stimuli. As such, disruption of this interaction results in a cellular metabolic switch promoting lactate production and enhanced capacity for tumorigenesis.

## Results

### Identification of Y260 as a Src-specific phosphorylation site

**Figure 1:**
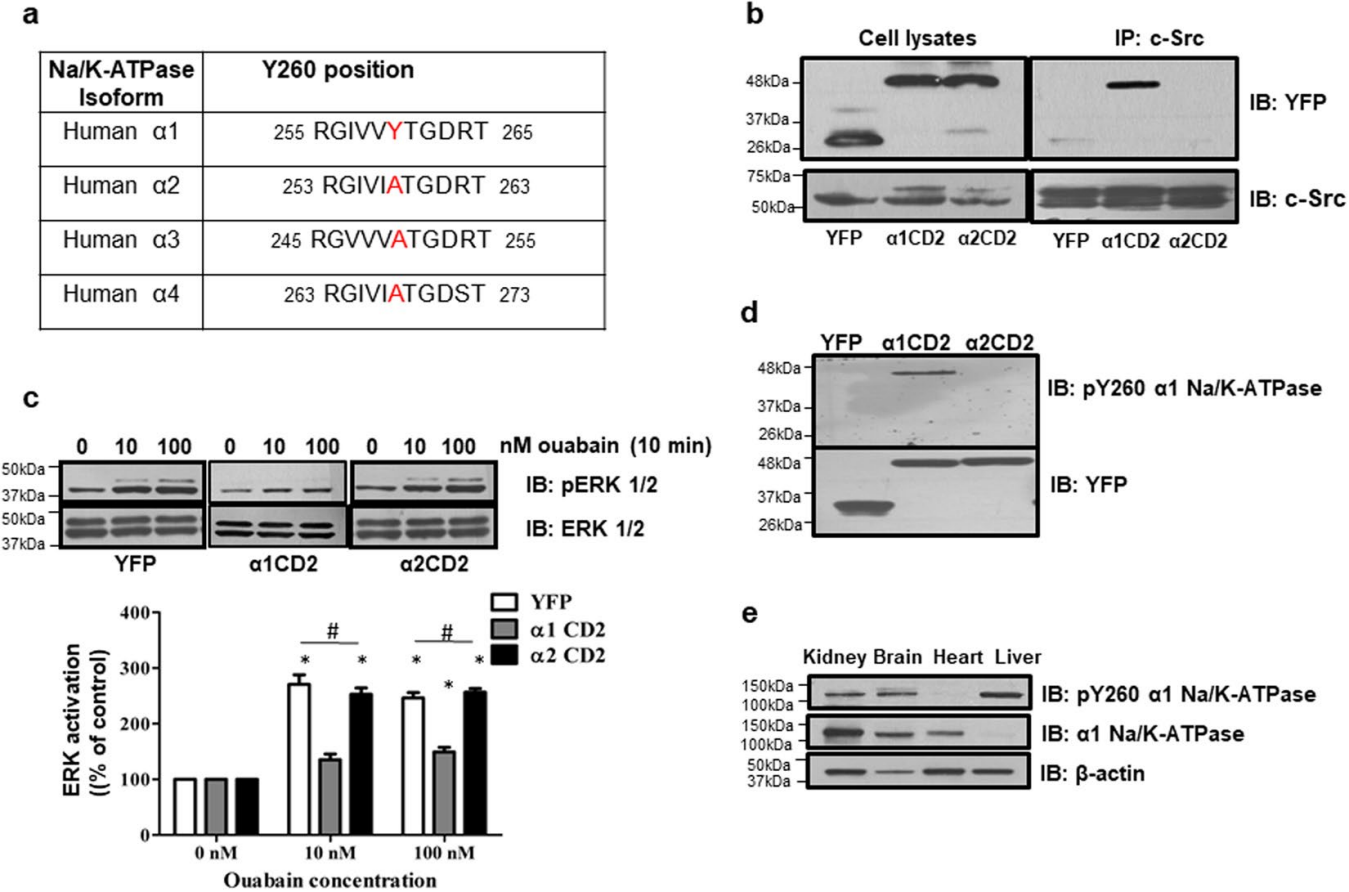
Identification of Y260 in α1 Na/K-ATPase as the major Src binding site- **(a)** Comparison of Y260 containing sequences in second cytoplasmic domain (CD2) of different human Na/K-ATPase isoforms. **(b)** Interaction between CD2 and Src in different cell lines. Representative blots are shown, n = 3. **(c)** Effects of ouabain on ERK activation. *p < 0.05 compared with vehicle-treated control of the same cell line (Student’s T-test). ^#^p < 0.05 compared between different cell lines (One-way ANOVA). n = 4. **(d)** Detection of Y260 phosphorylation in CD2. Representative blots are shown, n = 3. **(e)** Y260 phosphorylation and expression of α1 Na/K-ATPase in mouse tissues. A representative blot is shown, n = 3.

We have demonstrated that the second cytosolic domain (CD2) of α1 Na/K-ATPase functions like a Src SH2 domain ligand^15^. By comparing amino acid sequences of CD2 from different α isoforms, we found that α1 CD2, but not CD2 from other isoforms, contains a Tyrosine (Y260) residue (Fig. 1a). To test the potential role of Y260 in α1 isoform-specific interaction with Src kinase, we did the following studies.

We generated stable cell lines expressing either YFP-α2 CD2 or YFP-α1 CD2 (Supplementary Fig. 1A) and show that α1 CD2, but not α2 CD2 or control YFP, co-immunoprecipitated with Src from cell lysates (Fig. 1b). Consistent with our previous findings^15^, the expression of α1 CD2 blocked ouabain (a Na/K-ATPase-specific ligand)-induced ERK activation, whereas α2 CD2 failed to do the same (Fig. 1c). Similarly, data presented in Supplementary Fig. 1B–D show that the expression of Y260 containing C-terminal, but not the N-terminal, half of CD2 was sufficient to block ouabain-induced signal transduction.

It is well known that Src SH2 domain binds phosphorylated tyrosine residues^18^. Therefore we tested whether Y260 could be phosphorylated. Y260 phosphorylation was detected in α1 CD2 cells as well as full-length α1 Na/K-ATPase in mouse tissue lysates using an anti-pY260 (phosphorylated tyrosine 260) Na/K-ATPase α1 antibody (Fig. 1d,e). Interestingly, the level of pY260 varied among different tissues with the lowest being expressed in the heart, and the highest in the liver where the total expression of α1 Na/K-ATPase is the lowest among the examined tissues.

**Figure 2:**
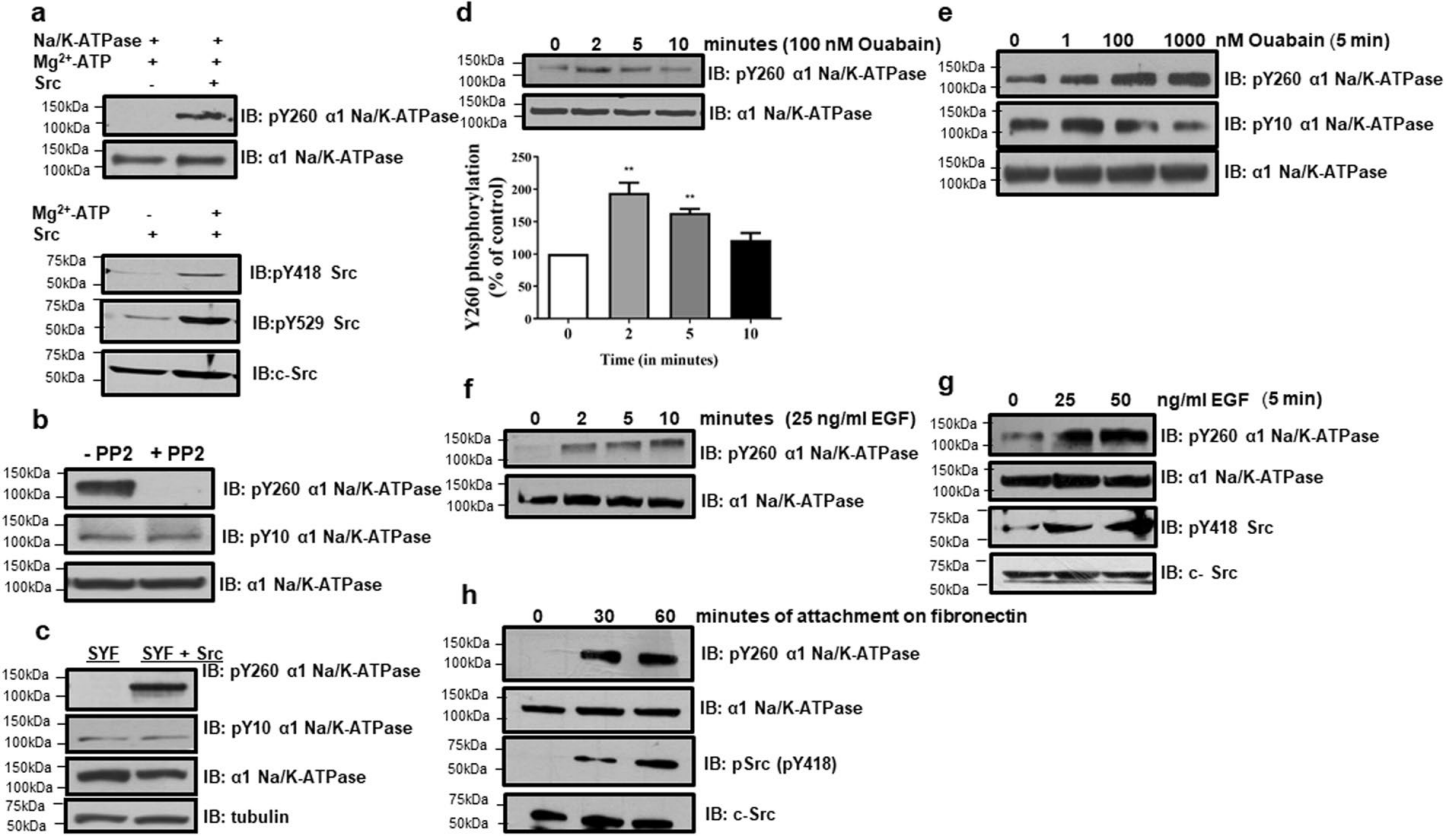
Y260 phosphorylation and Src- **(a)** Y260 phosphorylation in purified pig kidney α1 Na/K-ATPase (2 µg) by Src (4.5 units) in presence of 2 mM Mg^2+^-ATP in 10 minutes. Representative blots are shown, n = 3. Control blots showing Src phosphorylation at Y418 and Y529 sites by Mg^2+^-ATP are shown. **(b)** Effects of PP2 (5 μM, 30 minutes) on tyrosine phosphorylation of α1 Na/K-ATPase in LLC-PK1 cells. A representative immunoblot is shown. n = 4–5. **(c)** Effects of Src family kinase knockout on Y260 phosphorylation. Cell lysates were prepared from Src, Yes, Fyn knockout SYF, and Src-rescued SYF cells. Representative blots are shown, n = 4. **(d)** Effects of ouabain on Y260 phosphorylation as a function of time in LLC-PK1 cells. **p < 0.01 compared with vehicle-treated control (One-way ANOVA), n = 3. **(e)** Effects of different concentrations of ouabain on Y260 phosphorylation in LLC-PK1 cells. **(f,g)** Effects of recombinant EGF on Y260 phosphorylation in LLC-PK1 cells on time-**(f)** and dose-**(g)** dependent manner. Representative immunoblots are shown. n = 3. **(h)** Effects of integrin signaling on Y260 phosphorylation. Cell attachment-induced integrin signaling was measured by plating cells on fibronectin-coated dishes as described in “Methods”. Representative blots are shown. n = 3.

To identify the responsible protein for this phosphorylation, GST-tagged α1 CD2 was incubated with purified Src in the presence of ATP and probed for Y260 phosphorylation using the same anti-pY260 antibody. Phosphorylation was detected in GST-CD2 but not in GST (Supplementary Fig. 1E). Similarly, Src was able to phosphorylate purified pig kidney α1 Na/K-ATPase at Y260 as well (Fig. 2a). Finally, pretreatment of LLC-PK1 cells with PP2, a Src family kinase inhibitor, attenuated Y260 phosphorylation (Fig. 2b). We further verified our findings using cell lysates from SYF (Src family knock out) and Src-rescued SYF cells^19^. Y260 phosphorylation of α1 Na/K-ATPase was detected in Src-rescued, but not in parental, SYF cells (Fig. 2c). To address the specificity of this regulation, we also measured Y10 phosphorylation in α1 Na/K-ATPase that is mediated by insulin signaling^20^. Y10 phosphorylation was independent of Src kinase (Fig. 2b,c). These data indicate that Y260 is a Src-specific phosphorylation site and that the anti-pY260 antibody specifically recognizes both truncated and full-length α1 Na/K-ATPase polypeptide when they are phosphorylated at Y260.

### Y260 phosphorylation represents a general feature of Src signaling

If α1 Na/K-ATPase represents an important general regulator of Src, we would expect an increase in Y260 phosphorylation when cells are stimulated not only by ouabain but also other receptor ligands. We therefore tested Y260 phosphorylation in three different signaling pathways where Src is necessary for signal transduction. First, ouabain increased Y260 phosphorylation in both time and concentration-dependent manner in LLC-PK1 cells (Fig. 2d,e). To ensure that this effect was Y260-specific, Y10 phosphorylation was also probed. Consistent with a previous study^20^, ouabain showed no effect on Y10 phosphorylation (Fig. 2e). Second, Y260 phosphorylation was stimulated by EGF. EGF, like ouabain, produced a time- and dose-dependent stimulation of Y260 phosphorylation of Na/K-ATPase as well as Src at Y418 (Fig. 2f,g). Furthermore, we plated cells on to fibronectin-coated plates and analyzed the stimulation of Y260 phosphorylation in integrin signaling pathway^21^. An increase in Y260 phosphorylation was noted and correlated well with the activation of Src (Fig. 2h). These data indicate that Y260 phosphorylation represents a general feature of Src regulation, not only relevant to receptor function of Na/K-ATPase but also to receptor tyrosine kinases and integrin signaling.

### Y260 is required for Src-mediated signaling

To investigate the role of Y260 in Src-mediated signal transduction, we generated a stable cell line that expresses a loss of function (Y260A) mutant rat α1 Na/K-ATPase. An α1 Na/K-ATPase knockdown cell line, PY-17 was used for transfection^7^. A normal rat α1 Na/K-ATPase-rescued cell line, AAC-19, was used as a control. Control experiments (Supplementary Fig. 2A) show that the clone 21 and AAC-19 expressed comparable amount of rat α1. ^3^H-ouabain binding assays indicate that the expression of endogenous pig α1 Na/K-ATPase in Y260A mutant cells only amounted to about 1% of the total Na/K-ATPase (Supplementary Fig. 2D)^13,22^. Functionally, the expressed Y260A mutant was expressed in the plasma membrane, fully capable of rescuing the expression of β1 subunit and forming a functional Na/K-ATPase exhibiting comparable ouabain-sensitive ATPase activity as in AAC-19 cells (Supplementary Fig. 2B–E).

**Figure 3:**
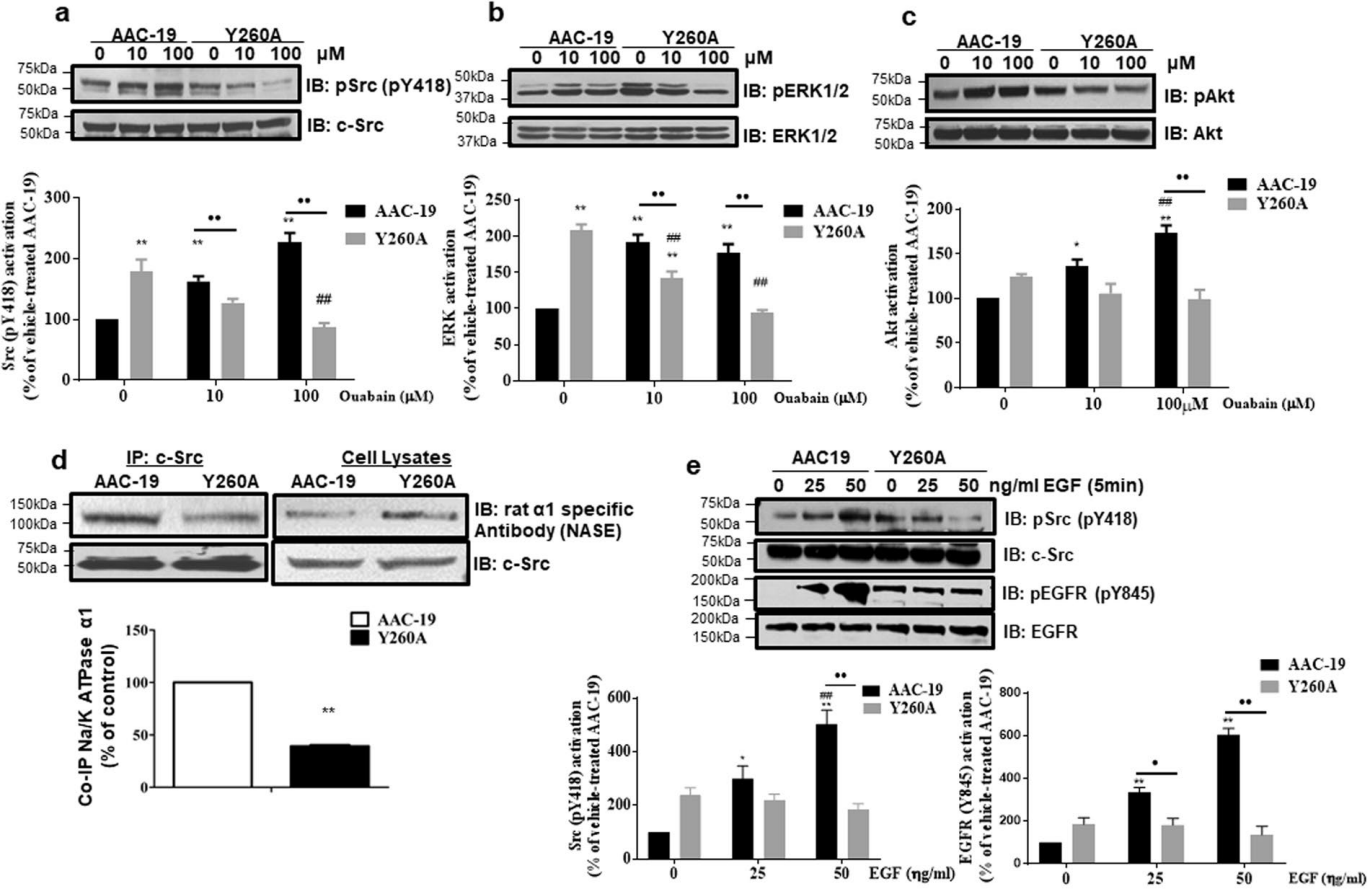
Effects of Y260A mutation on Src-mediated signal transduction- **(a**–**c)** Effects of Y260A mutation on ouabain-induced signaling. AAC-19 (control) and Y260A mutant cells were treated with different concentrations of ouabain for 10 minutes. Cell lysates were collected and analyzed for Src activation (pY418Src/c-Src) **(a)**, ERK activation (pERK1/2/ERK1/2) **(b)** and Akt activation (pS473Akt/Akt) **(c)**. *p < 0.05 and **p < 0.01 compared with vehicle-treated AAC-19 control, ^##^p < 0.01 compared with vehicle-treated Y260A control, and ^●●^p < 0.01 between different cell lines in the same treatment dose as indicated (Two-way ANOVA). n = 4–5. **(d)** α1 Na/K-ATPase/Src interaction analyzed by immunoprecipitation. **p < 0.01 compared with AAC-19 (Student’s T-test). n = 3. **(e)** Effects of Y260A mutation on EGF signaling assessed by Src activation and EGFR activation. Representative blots and quantifications are shown. n = 3. The same statistical symbols are used as in (**a**–**c**).

Next, we measured the effects of Y260A mutation in two different signaling pathways where Src plays an important role. First, Y260A mutant and AAC-19 cells were treated with different concentrations of ouabain, and subjected to Western blot measurements of Src, ERK, and Akt activities. Ouabain stimulated Src, ERK and Akt phosphorylation in AAC-19 cells. These stimulations were abolished by the Y260A mutation (Fig. 3a–c). To confirm that the observed effect was due to the inhibition of Na/K-ATPase/Src interaction, we conducted immunoprecipitation analysis. Y260A mutation resulted in a significant decrease (~60%) in the binding of α1 Na/K-ATPase to Src as compared to AAC-19 cells (Fig. 3d). Control experiments show that another clone of Y260A mutant cells, clone 24, like clone 21, also failed to respond to ouabain stimulation (Supplementary Fig. 3A,B).

Second, we assessed the role of Y260 in the EGFR signaling. Y260A mutant clone 21 and AAC-19 cells were exposed to different concentrations of EGF and measured for EGFR phosphorylation. As depicted in Fig. 3e, EGF stimulated Src kinase activity in AAC-19, but not in Y260A mutant cells. In accordance, EGF stimulated the phosphorylation of EGFR at Y845, a known Src-phosphorylation site^23^, in AAC-19, but not in Y260A mutant cells. Interestingly, we observed that the basal EGFR Y845 phosphorylation was significantly increased in Y260A mutant cells, indicating an important role of Y260-mediated Src interaction in the regulation of basal Src and Src-effector activity.

To corroborate this finding, we first measured the basal protein tyrosine phosphorylation. As shown in Supplementary Fig. 2F, Y260A cells expressed more tyrosine-phosphorylated proteins. To test whether this effect was Src-mediated, we measured Src phosphorylation at Y418 and Y529. As shown in Supplementary Fig. 2F, Src activity (pY418) was increased almost 2 folds in Y260A cells but there was no change in Y529 phosphorylation. Consistently, basal ERK activities were increased in Y260A cells in comparison to those in AAC-19 cells (Fig. 3b), which was sensitive to PP2, a Src inhibitor (Supplementary Fig. 2G).

### Y260A mutation leads to metabolic switch

The above studies indicate that Y260 phosphorylation is a major regulatory mechanism of Src-mediated signal transduction in the plasma membrane in response to a variety of stimuli. To assess the general significance of this newly discovered signaling mechanism, we probed a potential role of Y260 in the regulation of cellular metabolism.

**Figure 4:**
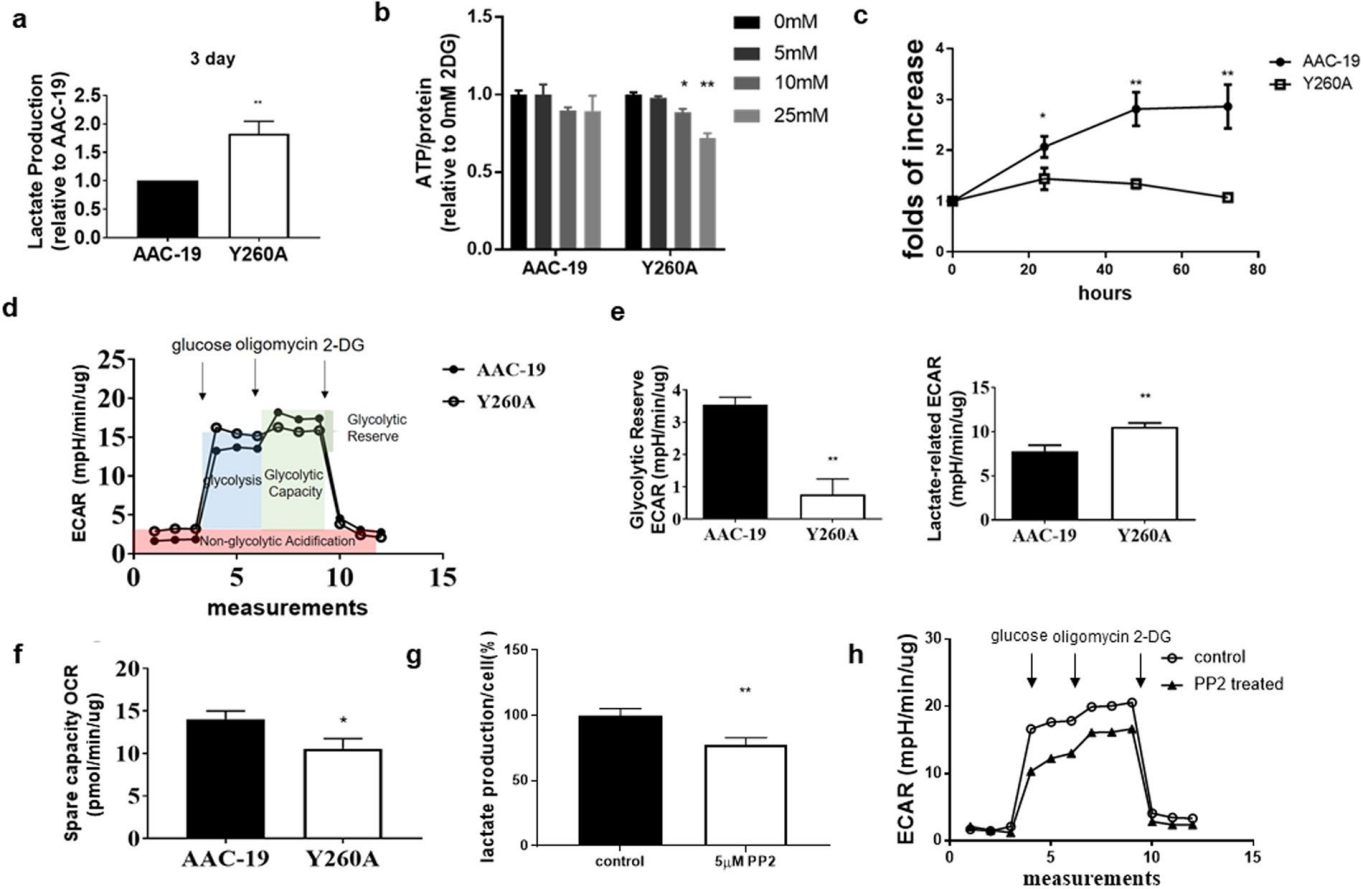
Effects of Y260A mutation on cellular metabolism- **(a)** Lactate production from culture media of AAC-19 and Y260A mutant cells. **p < 0.01 compared with control AAC-19 cells, n = 3. **(b)** Effects of different concentrations of 2-DG on cellular ATP content. *p < 0.05 and **p < 0.01 compared with vehicle-treated control (same cell line) (One-way ANOVA). **(c)** Effects of glucose removal on cell growth. Cell number was counted and presented as folds of change. *p < 0.05 and **p < 0.05 compared with 0 hour in the same cell line (One-way ANOVA), n = 3–4. **(d)** Bioenergetic parameter ECAR measurements of AAC-19 and Y260A cells. An actual representative trace of 4–7 separate measurements is presented. **(e)** Glycolytic reserve capacity and lactate-related ECAR were calculated from the experiments presented in **d** as marked. n = 4–7. **p < 0.01 compared with AAC-19 cells (Student’s T-test). **(f)** Bioenergetic parameter-OCR measurements of AAC-19 and Y260A mutant cells. Spare capacity was calculated from OCR measurements as described in Methods. *p < 0.05 compared with AAC-19 cells (Student’s T-test), n = 3–5. **(g)** Lactate production from culture medium of Y260A cells in the presence or absence of 5 μM PP2 for 4 h. **p < 0.01 compared with Y260A control. **(h)**ECAR measurement in Y260A cells cultured in the presence or absence of 5 μM PP2 for 4 h.

In the routine culture of Y260A mutant cell lines (both clone 21 and 24), we noticed faster acidification of culture medium than that of control AAC-19 cells. Measurement of medium confirmed that Y260A mutant cells produced 80% more lactate than that of AAC-19 cells (Fig. 4a). This suggests that disruption of Y260-mediated Src interaction may cause a metabolic switch from mitochondrial oxidative phosphorylation to aerobic glycolysis. To further test this working hypothesis, we first measured cellular ATP content in response to the inhibition of glycolysis by 2-deoxyglucose (2-DG). Compared to AAC-19 cells, Y260A cells were much more sensitive to 2-DG (Fig. 4b). Second, we measured the effect of glucose depletion on cell growth. Y260A mutant cells failed to grow in the absence of glucose whereas AAC-19 cells grew. In fact, the number of AAC-19 cells more than doubled during this time (Fig. 4c).

Then we determined extracellular acidification rate (ECAR) in both AAC-19 and Y260A cells in the presence of different inhibitors^24^. Cells were first cultured in glucose-free basal medium. The addition of glucose to cells induced a large increase in ECAR, indicating an increase in aerobic glycolysis (Fig. 4d). To measure the maximal capacity of glycolysis, this was followed by the addition of oligomycin that inhibited mitochondrial ATP production, resulting in a further increase in ECAR in AAC-19, but not in Y260A cells. Moreover, the addition of glycolysis inhibitor 2-DG completely reversed the increase in ECAR. Data analyses indicated a diminished glycolytic reserve in Y260A mutant cells and an increase in aerobic glycolysis (Fig. 4e). When oxygen consumption rate was measured, no major defects in mitochondrial function (e.g. respiratory control ratio and coupling efficiency) were noted (Supplementary Fig. 4B,C) except that the reserve capacity was reduced in Y260A mutant cells (Fig. 4f). Because oncogene activation is known to change cellular metabolic phenotype^25–33^, we tested whether this effect was dependent on Src dysregulation by α1 Na/K-ATPase in Y260A cells. As depicted in Fig. 4g and h, Src inhibition by treatment of Y260A cells with PP2, a Src inhibitor, was sufficient to reduce both lactate production (Fig. 4g) and glycolysis rate (Fig. 4h).

To further investigate the metabolic adaptation, we compared the gene expression profiles of Y260A mutant cells and control AAC-19 cells by RNAseq analysis. Several important genes involved in the glycolytic metabolism were significantly upregulated in Y260A mutant cells (Supplementary Fig. 4D). Notably, hexokinase 2 isoform (HK2), pyruvate dehydrogenase kinase (PDK) and lactate dehydrogenase (LDHA) were significantly increased, which has been also observed in cancer cells that underwent metabolic switch^1^. We also observed a significant increase in the expression of glucose transporter GLUT4 and a few amino acid transporters in Y260A, providing further support to the notion that these mutant cells display a metabolic adaptation similar to cancer cells. To further confirm that these effects were mediated by the dysregulation of Src kinase, we treated Y260A cells with PP2 to see if it could reverse the change in metabolic gene expression. As shown in Supplementary Fig. 4E, PP2 treatment restored the mRNA level of most of these upregulated genes, as measured by qPCR.

### Y260 phosphorylation is reduced in many cancer cell lines

**Figure 5:**
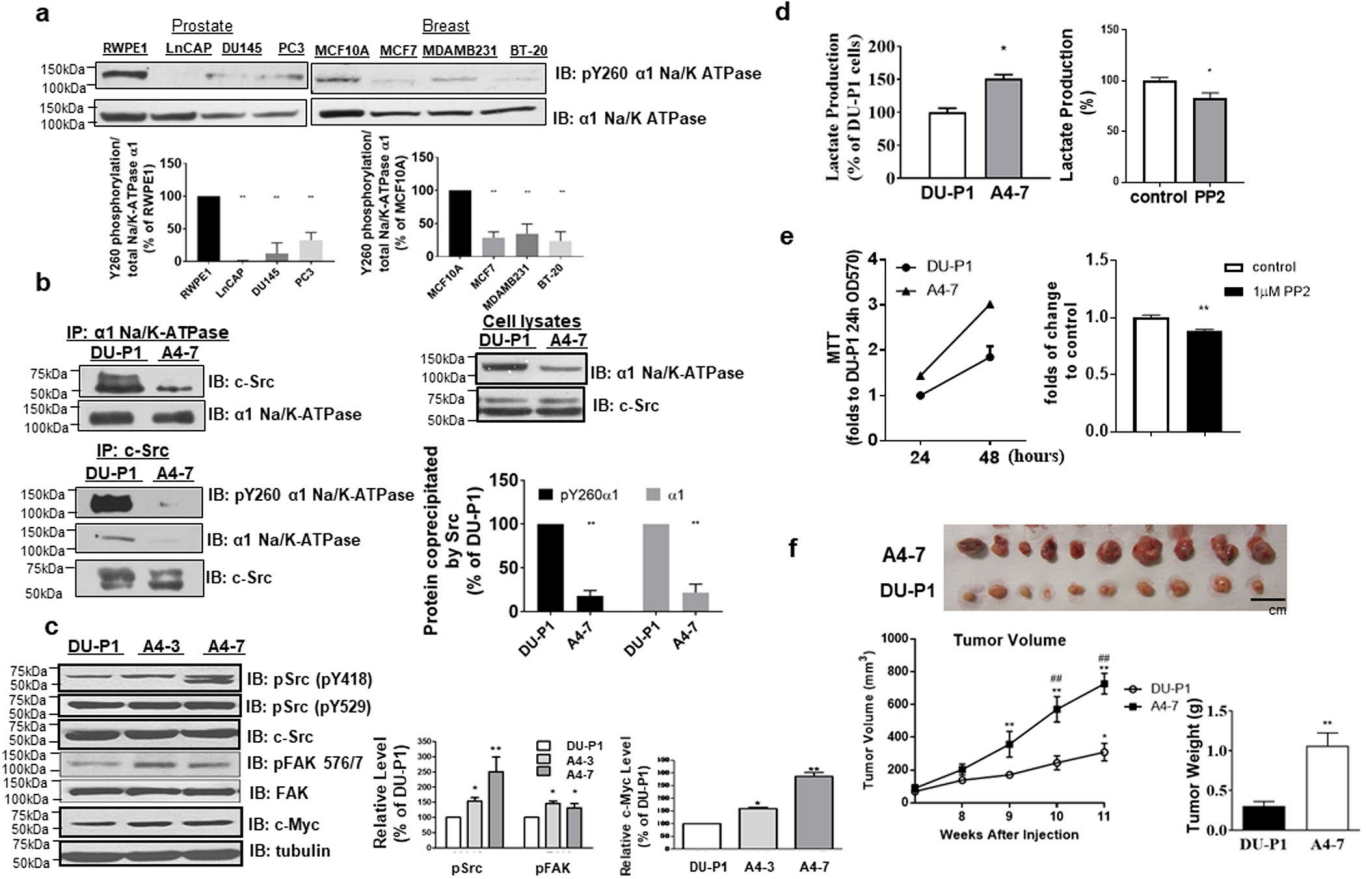
Y260 phosphorylation and α1 Na/K-ATPase/Src interaction in human cancers- **(a)** Measurement of Y260 phosphorylation in cancer cell lines. Cell lysates from a panel of prostate (LNCAP, DU145, and PC3) and breast (MCF7, MDAMB231, and BT-20) cancer cell lines were compared with corresponding control cells (prostate RWPE1 and breast MCF10A), for Na/K-ATPase α1 expression and Y260 phosphorylation. **p < 0.01compared with respective normal cell line (Unpaired T-test, Welch’s test), n = 3. **(b)** Interaction between Src and α1 Na/K-ATPase in control DU-P1 and knock-down A4–7 cells analyzed by immunoprecipitation. **p < 0.01 compared with DU-P1(Student’s T-test), n = 4. **(c)** Effects of α1 Na/K-ATPase knock-down on Src activity, FAK activity and Myc, analyzed by immunoblots. Representative immunoblots are shown and quantitative data from 4–5 separate experiments are presented. *p < 0.05 and **p < 0.01 compared with DU-P1 (Student’s T-test). **(d)** Measurement of lactate in medium from DU-P1 and A4-7 cell culture (left panel) and in medium from A4-7 cells cultured in the presence or absence of 5 μM PP2 for 4 h (right panel). *p < 0.05 compared with DU-P1 (left panel, n = 4) or A4-7 without PP2 treatment (right panel, n = 3) (Student’s T-test). **(e)** Cell proliferation rate of DU-P1 vs. A4-7 in 48 hours (left panel) and PP2 inhibition of A4-7 proliferation at 48-hour (right panel). **p < 0.01 compared with untreated control**. (f)** Effects of α1 Na/K-ATPase knock-down on tumor growth. DU-P1 and A4-7 cells were xenografted into NOD/SCID mice and tumor growth was assessed by measuring the tumor volume at different time points. Photos of tumors harvested from xenografted mice are shown in the upper panel. Quantitative data of tumor weight and volume from xenografted DU-P1 and A4-7 cells are presented in the lower panel. *p < 0.05 and **p < 0.01 compared with basal tumor volume, ^##^p < 0.01 compared between DU-P1 xenograft and A4-7 xenograft at the same time point (Two-way ANOVA), and **p < 0.01 compared with DU-P1 xenograft weight (Student’s T-test), n = 10.

In view of the important role of metabolic switch in cancer biology, the above findings led us to hypothesize that cancer cells may lose the capacity of α1 Na/K-ATPase-mediated Src regulation. To test this hypothesis, we compared the pY260 level in two different panels of cancer cell lines, which should be an indicator of Na/K-ATPase/Src interaction. As shown in Fig. 5a, Y260 phosphorylation was greatly reduced in both prostate and breast cancer cell lines in comparison to the corresponding control cell lines. To verify this, we immunoprecipitated Src kinase in selected cell lines and compared the co-precipitated α1 Na/K-ATPase level between cancer and control cell lines. We detected a significant decrease in Na/K-ATPase/Src interaction by co-immunoprecipitation (Supplementary Fig. 5A).

### Knocking down of α1 Na/K-ATPase increases aerobic glycolysis in DU145 cells and promotes the growth of tumor xenograft

To test whether α1 Na/K-ATPase-mediated Src interaction is important for control of aerobic glycolysis and tumor growth, we knocked down α1 Na/K-ATPase expression in DU145 prostate cancer cells using siRNA and generated stable cell lines A4-7, A4-3 and a control vector-transfected DU-P1 cell line. The DU-P1 cells expressed similar amount of α1 Na/K-ATPase like parental DU145 cells whereas A4-7 cells showed about 50% down-regulation (Supplementary Fig. 5B). Functional analyses indicate that knockdown of α1 Na/K-ATPase caused a significant decrease in Na/K-ATPase/Src interaction (Fig. 5b). A significant increase in Src and its effector FAK (Focal Adhesion Kinase) activity was noted in A4-7 cells (Fig. 5c). Moreover, EGF was able to stimulate Src activation and Y845 phosphorylation of EGFR in control DU-P1 cells but not in A4-7 cells (Supplementary Fig. 5C). Like Y260A mutant cells, basal EGFR Y845 phosphorylation was increased in A4-7 cells. Moreover, A4-7 cells expressed higher amount of Myc, which is indicative of their more aggressive and proliferative nature (Fig. 5c)^34^. In accordance, we detected a further increase in lactate production in A4-7 cells, which was sensitive to Src inhibition by PP2 (Figs 5d and Supplementary Fig. 5E). To compare the tumor forming ability, we tested the cell proliferation rate of A4-7 cells against DU-P1 cells. As expected the cell proliferation rate of A4-7 was significantly higher than that of the control, and reversed by PP2 (Fig. 5e). Finally, when control DU-P1 and A4-7 cells were implanted into NOD/SCID mice, we observed a close to four-fold increase in tumor size of A4-7 vs DU-P1 (Fig. 5f).

### Expression of α1 Na/K-ATPase is reduced in several human cancers, especially the metastatic lesions

**Figure 6:**
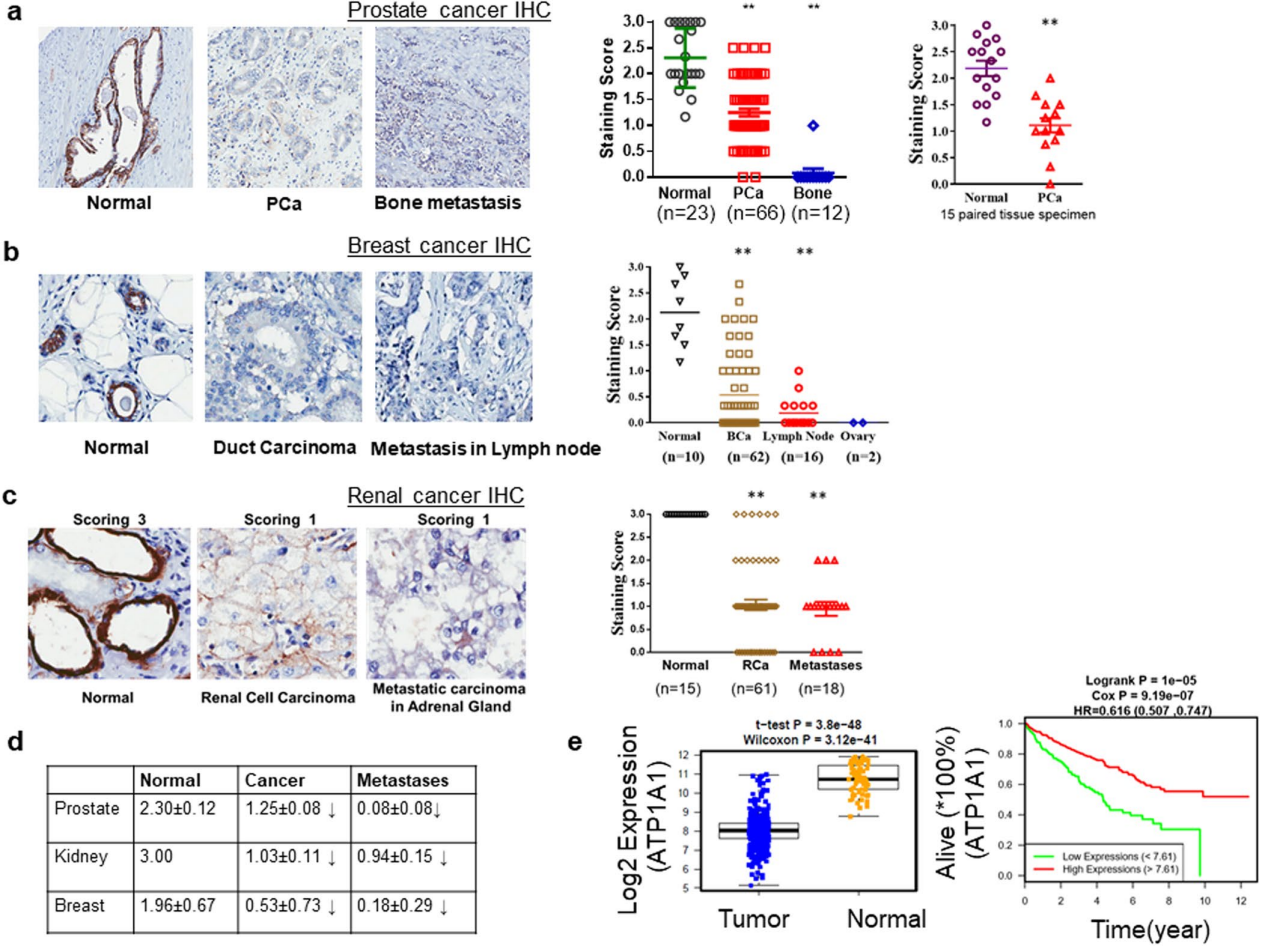
Measurement of α1 Na/K-ATPase expression in primary tumor and metastatic lesions- **(a)** The expression of α1 Na/K-ATPase in prostate cancer. Left panels show α1 expression patterns in paired human normal prostate tissue (left), carcinoma (middle) and bone metastasis (right). Human tissue arrays were immunostained with a α1 monoclonal antibody (in brown). Hematoxylin was used for counterstaining of cell nucleus (in blue). Quantitative data are shown on the right side. **p < 0.0001 (one-way ANOVA, Bartlett’s test). Down-regulation of α1 Na/K-ATPase in prostate cancer was further verified by paired tissue analysis (right-most panel) **p < 0.001, paired T-test (Wilcoxon signed-rank test). **(b,c)** The expression of α1 Na/K-ATPase in breast and kidney cancers. Left panels show α1 Na/K-ATPase expression patterns in normal tissues, cancer and metastatic lesions as in A. Right panel shows quantitative data of α1 staining. **p < 0.0001 (one-way ANOVA, Bartlett’s test). **(d)** Comparison of α1 Na/K-ATPase expression in three different human cancers. Quantitative measurements of Na/K-ATPase α1 expression in Panel a-c are tabled. **(e)** Transcriptional down-regulation of Na/K-ATPase α1 (ATP1A1) gene expression and kidney cancer patient survival. The left panel shows a decrease in α1 expression in human kidney cancer (TCGA-KIRC database, n = 530), paired T-test (Wilcoxon signed-rank test). The right panel shows an inverse correlation between the α1 gene expression and patient survival (log-rank survival test).

To assess the clinical relevance of our new findings, we measured α1 protein expression in three different types of human cancer where the expression of α1 Na/K-ATPase in the normal epithelium is high. First, we analyzed 66 prostate carcinoma, 12 bone metastatic and 23 normal tissue samples. The expression of α1 Na/K-ATPase was significantly reduced (Fig. 6a) in prostate carcinoma (n = 66) vs control (n = 23). This was confirmed by paired analyses (normal vs carcinoma, n = 15). Most importantly, there were no detectable α1 Na/K-ATPase signals in 11 out of 12 bone metastatic samples. Second, we compared 10 normal breast tissue and 62 ductal carcinoma samples, and corresponding metastatic samples in the ovary and lymph node (Fig. 6b). The expression of α1 Na/K-ATPase was significantly decreased in the primary tumor and further reduced in metastatic samples. Third, a significant decrease in α1 Na/K-ATPase expression was also found in renal cell carcinoma (n = 61) in comparison with the control (n = 15) (Fig. 6c). Interestingly, a decrease in adrenal gland metastasis of renal carcinoma appeared to be less severe than that in bone lesions of prostate cancer. However, α1 Na/K-ATPase was highly expressed in normal kidney tubules (15 out of 15 scored 3 in normal tissue). This level of expression was detected in only about 10% of renal cell carcinoma (6 out of 61) and further reduced to 0% in metastatic lesions.

Transcriptional regulation could represent an important mechanism of changing α1 expression in human cancers. Database search reveals that the expression of α1 Na/K-ATPase mRNA was significantly reduced (p < 0.001) in renal clear cell carcinoma in the TCGA-KIRC database (n = 530). The mean log 2 values are 11 and 8.4 for the normal kidney and renal carcinoma, respectively. Most importantly, this expression pattern inversely correlated with patient survival rate, with lower expression being associated with a high mortality rate (Fig. 6e). A significant decrease in the expression of α1 Na/K-ATPase mRNA in prostate cancer was also detected. However, the difference was less than 0.3 Log 2 value. As such, there was no correlation between lowered mRNA expression and patient survival rate in prostate cancer (Supplementary Fig. 6A,B).

## Discussion

**Figure 7:**
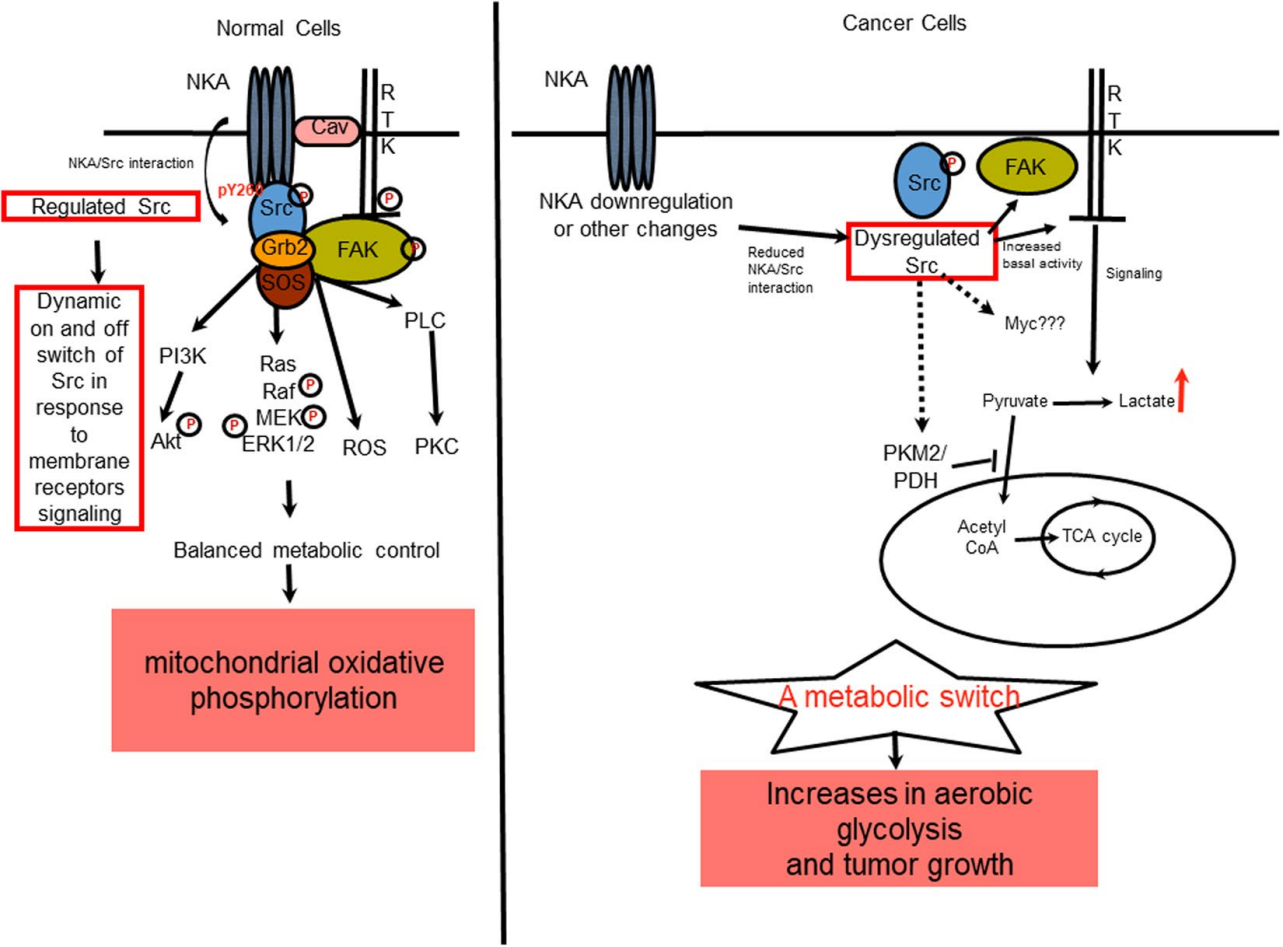
Schematic diagrams of α1 Na/K-ATPase-mediated Src regulation in normal and cancer cells. Established signaling pathways are denoted by solid black arrows and speculated signaling pathways are denoted by broken black arrows. NKA = Na/K-ATPase, Cav = Caveolin 1, FAK = Focal Adhesion Kinase, RTK = Receptor Tyrosine Kinase, PI3K = Phosphatidyl Inositol 3 Kinase, ROS = Reactive Oxygen Species, PLC = Phospholipase C, PKC = Protein Kinase C, PKM2 = Pyruvate Kinase isoenzyme M2, PDH = Pyruvate Dehydrogenase, ERK = Extracellular Regulatory Kinase. The circled letter P denotes phosphorylation and activation.

We report here the discovery of α1 Na/K-ATPase Y260 as a Src-specific phosphorylation and binding site, and an increase in Y260 phosphorylation as a general feature of Src-mediated signal transduction in response to the activation of membrane receptors. It appears that this dynamic regulation of Src by α1 Na/K-ATPase is significantly attenuated or lost in human cancer cells. This dysregulation increases the basal Src activity, resulting in the activation of Src effectors such as ERK, EGFR, Akt and FAK, all of which are implicated in cancer progression^35–38^. Furthermore, we provide evidence that the loss of Src regulation by α1 Na/K-ATPase causes a metabolic switch and promotes the formation and growth of tumor xenograft. A schematic diagram of α1 Na/K-ATPase-mediated Src regulation and the consequence of the loss of this regulation is given in Fig. 7. These and other important issues are further discussed.

### Na/K-ATPase α1 isoform as an underappreciated albeit important regulator of Src family kinases

Recent studies have indicated an important role of α1 Na/K-ATPase-mediated and Src-dependent pathways in control of embryonic development, renal salt handling and in disease progression where inflammation and ROS stress play an important role^39–43^. Interestingly, it appears that α1 Na/K-ATPase also interacts with other members of Src family kinases and that such interaction (e.g., Lyn) is a key to CD36- and CD40-mediated signal transduction^44–46^. The new findings presented here reaffirm the ability of α1 Na/K-ATPase to bind and regulate Src. Specifically Y260 and its phosphorylation by Src kinase appears to be very significant in many Src kinase mediated signaling pathways, since it participated in not just ouabain, but also EGF as well as integrin signaling. These data indicate that α1 Na/K-ATPase is an essential partner (regulator) of Src kinase in the plasma membrane, which is further supported by the loss of function Y260A mutant studies. Moreover, the Y260A mutant Na/K-ATPase is fully functional as an ion pump, but defective in binding and regulating Src. As such, it blocks not only ouabain but also EGF-induced Src signaling. Moreover, it also caused Src dysregulation as basal Src activity increased by almost 2 folds in Y260A cells. Concomitantly, we detected an increase in activity of ERK, which was sensitive to Src inhibitor, PP2 (Supplementary Fig. 2G). These findings are consistent with our prior observations that either knockdown of α1 Na/K-ATPase or expression of Src binding-α1 mutants increases basal Src and consequently ERK activity^13,16^. Thus, the new findings reaffirm our contention that the α1 Na/K-ATPase is a major regulator of the plasma membrane pool of Src. We further suggest that the loss of interaction between α1 Na/K-ATPase and Src will result in a dys-regulation of Src, which could lead to 1) the inability of ouabain-induced signaling and 2) increased basal Src activity.

Although many proteins interact and regulate Src, to the best of our knowledge, α1 Na/K-ATPase appears to be the only one that can regulate Src simultaneously through its two domain-domain interactions^11–13^. This balanced and sequential regulation is essential for the dynamic nature of Src signaling^47^. Disruption of either interaction alters Src signaling regardless of whether basal Src activity is increased or decreased^13,15,22^, resulting in changes in cellular metabolism and growth.

### The role of α1 Na/K-ATPase in the regulation of aerobic glycolysis and its implication in cancer biology

Although mutations in oncogenes like KRAS or PI3K genes have been implicated in the metabolic switch observed in cancer cells, how dysregulations of the proto-oncogene SRC affects tumor growth is not clear^1^. Src family kinases (SFK) are frequently hyper-activated in cancers but activating mutations or chromosomal rearrangements in Src are relatively rare in nature^2^. The precise molecular mechanism underlying the defective Src regulation in human cancers remains to be resolved.

Interestingly, Y260A mutant cells undergo a metabolic switch from mitochondrial oxidative phosphorylation to aerobic glycolysis (i.e., the increase in lactate production), a phenomenon commonly seen in oncogenic cells. The expression of several important glycolytic genes is upregulated in these cells, which further supports the phenotype. Consistently, PP2 treatment not only reduced the expression of these up-regulated genes involved in glycolysis, but also blocked the increase in lactate production (Fig. 4g,h). In short, these studies provide a molecular insight of how α1 Na/K-ATPase may work as a regulator of cellular metabolism through Src^4,5^.

We further suggest that this mechanism may provide a novel link connecting Na/K-ATPase α1 subunit downregulation in cancer to defective Src regulation and henceforth altered cellular metabolism. We demonstrate here that cells harboring Y260A mutant Na/K-ATPase have increased aerobic glycolysis and lactate production. Y260 phosphorylation was significantly reduced in human cancer cell lines, indicating a loss of Src regulation by α1 Na/K-ATPase. This finding is further substantiated by our tissue array data that show a significant decrease in α1 Na/K-ATPase in three different types of human cancers. Others have also reported a reduction in Na/K-ATPase α1 expression in lung, skin and testicular cancers^48–51^. Apparently, both transcriptional (e.g., kidney) and post-transcriptional mechanisms (e.g., prostate) are involved in the down-regulation of α1 Na/K-ATPase. The post-transcriptional regulation appears to involve increased endocytosis of α1 Na/K-ATPase^52^.

The importance of α1 Na/K-ATPase in cancer biology was further substantiated by two additional observations. First, the expression of α1 Na/K-ATPase is further reduced in metastatic samples from prostate, breast cancer, and renal cell carcinoma. Src activity is higher in metastatic lesions relative to primary tumor tissues^53,54^. It is plausible that the decrease in the expression of α1 Na/K-ATPase diminishes the regulation of Src, leading to an increase in basal Src activity. This is supported by the data that Src and its effectors such as EGFR and FAK activity were further increased in α1 knockdown A4-7 cells (Fig. 5c and Supplementary Fig. 5C). Moreover, A4-7 cells had increased lactate production and c-Myc expression (Fig. 5c,d), which is indicative of their more aggressive status. This is reaffirmed by an almost four-fold increase in tumor size when xenografted into mice (Fig. 5f). This observation is consistent with knockdown studies reported with other Src regulators like Csk^55^. Second, data analysis of renal clear cell carcinoma in the TCGA-KIRC database (n = 530) reveals a significant decrease in the expression of α1 mRNA in patient samples. This decrease is inversely correlated with the survival of patients suffering from renal clear cell carcinoma.

In short, our new findings provide strong evidence that α1 Na/K-ATPase works like a tumor suppressor by regulating the cellular Src kinase. We suggest that this newly discovered signaling mechanism may be targeted for developing new cancer therapeutics, which is supported by our early studies^52^. Furthermore, we speculate that Y260 phosphorylation may be utilized as an indicator for Na/K-ATPase/Src interaction in cancers. This may be of particular importance in some cancer types where α1 Na/K-ATPase expression is reportedly high^56^. Needless to say, this hypothesis remains to be tested. Finally, it is important to note that although metabolic switch is well established to play a role in tumorigenesis, our data do not allow us to conclude that increases in tumorigenesis in A4-7 cells are due to increased glycolysis. Interestingly, we observed that this switch actually inhibits cell proliferation in Y260A cells (Supplementary Fig. 4A). This apparent discrepancy in cellular fate may be explained by the recent findings that suggests activation of oncogene in normal cells can induce them to enter a non-proliferative state called senescence, whereas oncogene activation in cancer cells can cause them to become hyper proliferative^57,58^.

## Materials and Methods

### Materials

Antibodies and their sources: monoclonal anti-Src antibody (B12), polyclonal anti-ERK1/2 (Extracellular Regulatory Kinase 1/2) antibody, monoclonal anti-phospho ERK1/2 antibody, goat anti-mouse IgG HRP and goat anti-rabbit IgG HRP secondary antibodies -Santa Cruz Biotechnology (Santa Cruz, CA). Anti-Src (Y418) rabbit polyclonal antibody -Invitrogen (Carlsbad, CA). Anti-Akt, anti-phospho-Akt (S473), anti-PKM2 and anti-phospho-PKM2 (Y105) rabbit antibodies, phosphoFAK576/7 and FAK rabbit antibodies -Cell Signaling Technologies (Danver, MA). Monoclonal anti-α1 Na/K-ATPase subunit antibody (α6f) -Developmental Studies Hybridoma Bank at The University of Iowa (Iowa City, IA). Monoclonal anti-Src (GD-11) antibody, polyclonal rabbit anti-α1 Na/K-ATPase (06-520), Protein-G-agarose beads for immunoprecipitation and PP2 -Millipore (Billerica, MA). Anti-GFP and anti-c-Myc rabbit polyclonal antibodies -Abcam (Cambridge, MA). Anti-phospho-α1 Na/K-ATPase (Y260) -Assay Biotech (Fremont, CA). Transfection kit (Lipofectamine 2000) was from Invitrogen. QuikChange mutagenesis kit was from Stratagene (La Jolla, CA). Polyclonal rat α1 specific-antibody (anti-NASE) was kindly gifted by Dr. Thomas Pressley (Texas Tech University, Lubbock, TX). All other reagents were purchased from Sigma-Aldrich (St. Louis, MO).

### Mice studies

Animal protocols were approved by the Institutional Animal Care and Use Committee (IACUC) of Marshall University and University of Toledo according to NIH guidelines. Male C57/BL6J mice (8–10 weeks old) were humanely sacrificed and different organs were frozen immediately for preparation of tissue lysates for immunoblots. Tumor xenografts were established by subcutaneous injection of 5 × 10^6^ DU-P1 or A4-7 cells into the left and right flanks of 6-week-old female NOD/SCID mice (Charles River). Tumor length (L) and width (W) were measured with calipers and tumor volume was estimated as V (L × W^2^)/2.

### Immunohistochemical (IHC) Staining Analysis of Human Samples

Na/K-ATPase α1 IHC staining was performed by US Biomax (Rockville, MD) using human kidney, prostate and breast tissue microarray, and was carried out as described before^52^. As such, it is not possible to record the details of the patients^52^. Antibody for IHC- mouse monoclonal anti- Na/K-ATPase α1 antibody (Millipore). Nucleus was counterstained with hematoxylin. Two independent investigators examined the staining intensity and scored each slide three times as defined: 0, absent; 1, weak; 2, moderate; 3, strong. A mean score was recorded.

### Cell culture and cell line generation

All cell lines were purchased from and maintained according to ATCC (Mannassa, VA). LLC- PK1 was serum starved before being used for signaling experiments. AAC-19, Y260A and DU145, A4-7 cell lines were cultured in the presence of 0.5% and 1% serum respectively for 24 hours before being used for the signaling experiments. LLC-PK1 cells were transfected (Lipofectamine 2000) with pEYFP-C1 vector containing α1 CD2, α2 CD2 or pEYFP-C1 empty vector. After verifying YFP expression visually, the cells were selected with 1 mg/ml G418 for one week. G418 resistant clones were selected and expanded. Cells were then cultured without G418 for at least three generations before being used for experiments. CD2N and CD2C cells were generated in similar manner. Y260A cells were generated by transfecting PY-17 cells with pRC/CMV-α1 AACm1 vector harboring mutation at Y260 and selected with ouabain (3 μM). Ouabain-resistant clones were isolated and expanded into stable cell lines. The cells were cultured for at least 3 generations without ouabain before being used for any experiment. DU-P1, A4-7 and A4-3 cell lines were generated by transfecting DU145 cells with a α1 Na/K-ATPase-specific siRNA containing vector and selecting with Puromycin as described above. All constructs were verified by DNA sequencing.

### Immunoblot, Immunoprecipitation and Immunostaining Analysis

Immunoblot assays were performed as described before^11^. Intensity of bands were quantified with ImageJ software (NIH). Immunoprecipitation was performed by adding 5 µg of anti-Src (Millipore Cat# 05-184) antibody to 500 µg of cell lysate (1 µg/µl concentration) or 8 µg of anti-α1 Na/K-ATPase antibody (Millipore Cat# 06-520) to 800 µg of cell lysate (1 µg/µl concentration). Precipitated proteins were then analyzed as described before^13^.

For immunostaining antibodies used - anti-α1 Na/K-ATPase antibody (Millipore Cat# 05-369) and Alexa Fluor 488-conjugated anti-mouse secondary antibody.

### Cell growth assay and MTT assay

Cell growth assay and MTT assay were performed as previously described^52,59^.

### Biochemical measurement of ATP and lactate

ATP measurements were performed using CellTiter-Glo Luminescent Cell Viability Assay kit. 10,000 cells per well were cultured in 96- well culture plate. After treatment with 2-DG at indicated concentrations in serum-free DMEM for 45 minutes, assay reagents were reconstituted and added into culture plate. Luminescent counts of the reactants were determined from an opaque-walled 96-well plate with a microplate reader. Lactate measurement was done by colorimetric methods as described by Barker^60^. In PP2 study, culture medium containing serum and 5 μM PP2 was replaced in culture dish and collected after 4 hours for measurements.

### Bioenergetics

Properties of cellular bioenergetics were characterized using Seahorse XFp Extracellular Flux Analyzer by measuring oxygen consumption rate (OCR) and extracellular acidification rate (ECAR) following the guideline provided by the manufacture. Prior to the start of Seahorse assay, optimal FCCP concentration for each cell line was determined by titration studies. Unless indicated otherwise, 10,000 cells per well were seeded with culture medium. Bicarbonate-free medium (Agilent Technologies) with different substrates (10 mM glucose, 2 mM glutamine and 1 mM pyruvate for Cell Mito Stress Test assays; 2 mM glutamine for Glycolysis Stress Test assays) was replaced one hour prior to the assay. Baseline OCR and ECAR rates were measured three times before various inhibitors, stimulants, substrates, or compounds were added through the drug delivery ports. In PP2 study, cells were pretreated with 5 μM PP2 for 4 h and then analyzed.

### ^3^H ouabain binding assay and ATPase Activity Assay

^3^H-Ouabain Binding Assay and ATPase Activity Assay were performed as previously described^59^.

### Cell-attachment induced integrin signaling

Cell-attachment induced integrin signaling were done as previously described^15^.

### RNAseq Analysis

RNAseq analysis was performed by BGI Tech, China. Briefly, RNA was extracted from cell lysates, treated with DNase 1 and magnetic Oligod(T) beads were used to isolate mRNA. mRNAs were then fragmented with fragmentation buffer and cDNAs were synthesized using them as templates. After agarose gel electrophoresis, suitable fragments were selected for PCR amplification as templates. During QC steps, Agilent 2100 Bioanalyzer and ABI StepOnePlus Real-Time PCR System were used for quantification and qualification of the sample library. The library was sequenced using HiSeqTM 2000 sequencer. Bioinformatic analysis was performed by deep analysis of gene expression.

### RNA Extraction, cDNA Synthesis and Quantitative PCR

Total RNA was isolated with the QIAGEN RNeasy Mini Kit. The same amount of total RNA was used for synthesizing first-stand cDNA with the SuperScript III First-Stand Synthesis SuperMix for qRT-PCR (ThermoFisher). The cDNA from each sample was used as a template for the quantitative PCR (Syber green) with Roche LightCycler® 480 Real-Time PCR System. All primers were synthesized by Integrated DNA Technologies (IDT). β-actin was used as internal control.

### Statistical analysis

Data were recorded as mean +/− SEM (Standard Error of Mean). Student’s T-test were used to measure differences between two individual groups and one-way analysis of variance (ANOVA) was used to measure differences between more than two groups. Two-way ANOVA was used to measure between more than two groups while each group contained more than one variables. One-way ANOVA (Bartlett’s test) or paired T-test were used to measure differences in the tissue array data, where appropriate. Paired T-test followed by Wilcoxon signed rank test were used to analyze gene expression data from TCGA database. Survival analysis was measured by log-rank survival test. p value less than 0.05 was considered as significant.

### Data availability

The authors confirm all relevant data and figures are included in the paper and/or its supplementary information files.

## Electronic supplementary material

Supplementary Data
