# Supplementary material for: Na/K-ATPase Y260 Phosphorylation–mediated Src Regulation in Control of Aerobic Glycolysis and Tumor Growth
Source: Sci Rep. 2018 Aug 17;8:12322. doi: 10.1038/s41598-018-29995-2 (PMC6098021; doi:10.1038/s41598-018-29995-2)
Supplement: Supplementary file 1 — Supplementary Data [file 41598_2018_29995_MOESM1_ESM.docx]

Na/K-ATPase Y260 Phosphorylation–mediated Src Regulation in Control of Aerobic Glycolysis and Tumor Growth

Moumita Banerjee^1#^, Xiaoyu Cui^1#^, Zhichuan Li^2^, Hui Yu ^1,^ ^3^, Liquan Cai^1^, Xuelian Jia^1^, Daheng He^4^, Chi Wang ^4^, Tianyan Gao^5^, and Zijian Xie^1*^

^1^ Marshall Institute for Interdisciplinary Research (MIIR), Marshall University, Huntington, West Virginia 25755, U.S.A, ^2^ Department of Physiology and Pharmacology and Medicine, University of Toledo College of Medicine, Toledo, Ohio 43614, U.S.A, ^3^ Present Address-Department of Pediatrics, Union Hospital, Tongji Medical College, Huazhong University of Science and Technology, Wuhan, Hubei, 430022 China, ^4^ Department of Cancer Biostatistics, Markey Cancer Research Center, University of Kentucky, Lexington, Kentucky 40536, U.S.A, ^5^ Department of Molecular and Cellular Biochemistry, Markey Cancer Research Center, University of Kentucky, Lexington, Kentucky 40536 U.S.A.

**^#^** These authors contributed equally.

***Corresponding author**. Email: [xiez@marshall.edu](mailto:xiez@marshall.edu); Phone: 3046963852

SUPPLEMENTARY INFORMATION


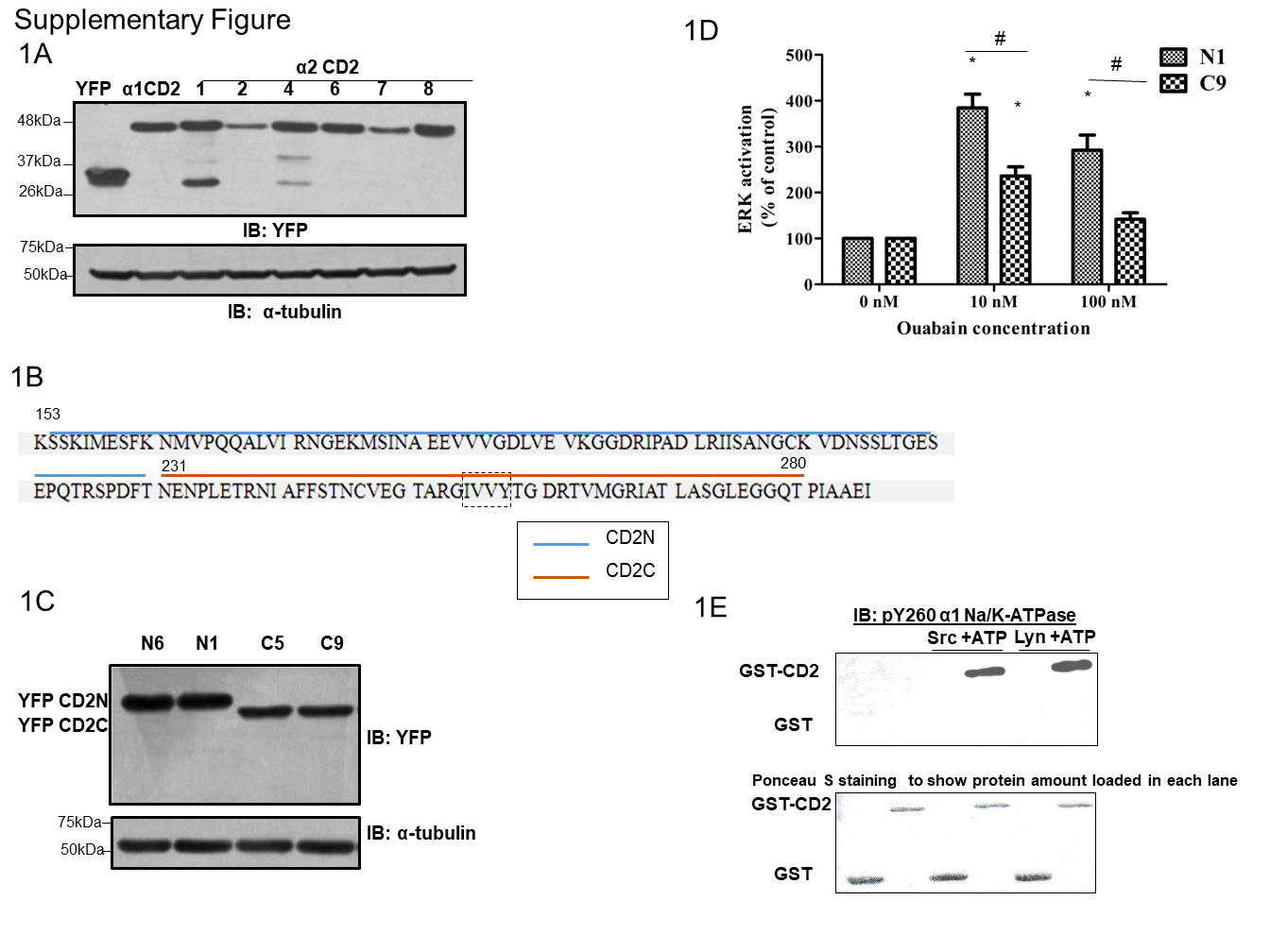


Supplementary Figure 1. (A) Expression of α1 and α2 CD2 in different cell lines. Different cell lines were generated as described in “Methods”. Cell lysates were prepared and subjected to Western blot using anti-GFP antibody. Representative blots are shown. n=3. (B) Amino acid sequence of CD2 (second cytoplasmic domain) of α1 Na/K-ATPase is shown. The N terminal fragment, denoted as CD2N, is labeled with a blue line and C terminal fragment, denoted as CD2C, is labeled with a red line. Predicted Src binding region is shown in a box. (C) Expression of CD2N and CD2C fragment in different cell lines. YFP-CD2N and YFP-CD2C cell lines were prepared as described in “Methods”. Cell lysates were made from each cell line as indicated, and subjected to Western blot as in A. Representative blots are shown, n=3. (D) Effects of ouabain on ERK activation. Cells were treated with ouabain as indicated, and cell lysates were made and subjected to Western blot analyses of ERK activation (pERK1/2/ERK 1/2). *p<0.05 compared with vehicle-treated control of the same cell line, #p<0.05 compared between different cell lines under the same treatment. n=4-5 (E) In vitro assay showing Y260 phosphorylation of purified GST or GST-α1 CD2 (5 µg) by purified recombinant Src or Lyn (4.5 units) in the presence of 2mM Mg^2+^-ATP. Upper panel shows pY260 α1 blot and lower panel shows Ponceau-S staining of the same membrane. Representative blots are shown, n=4.


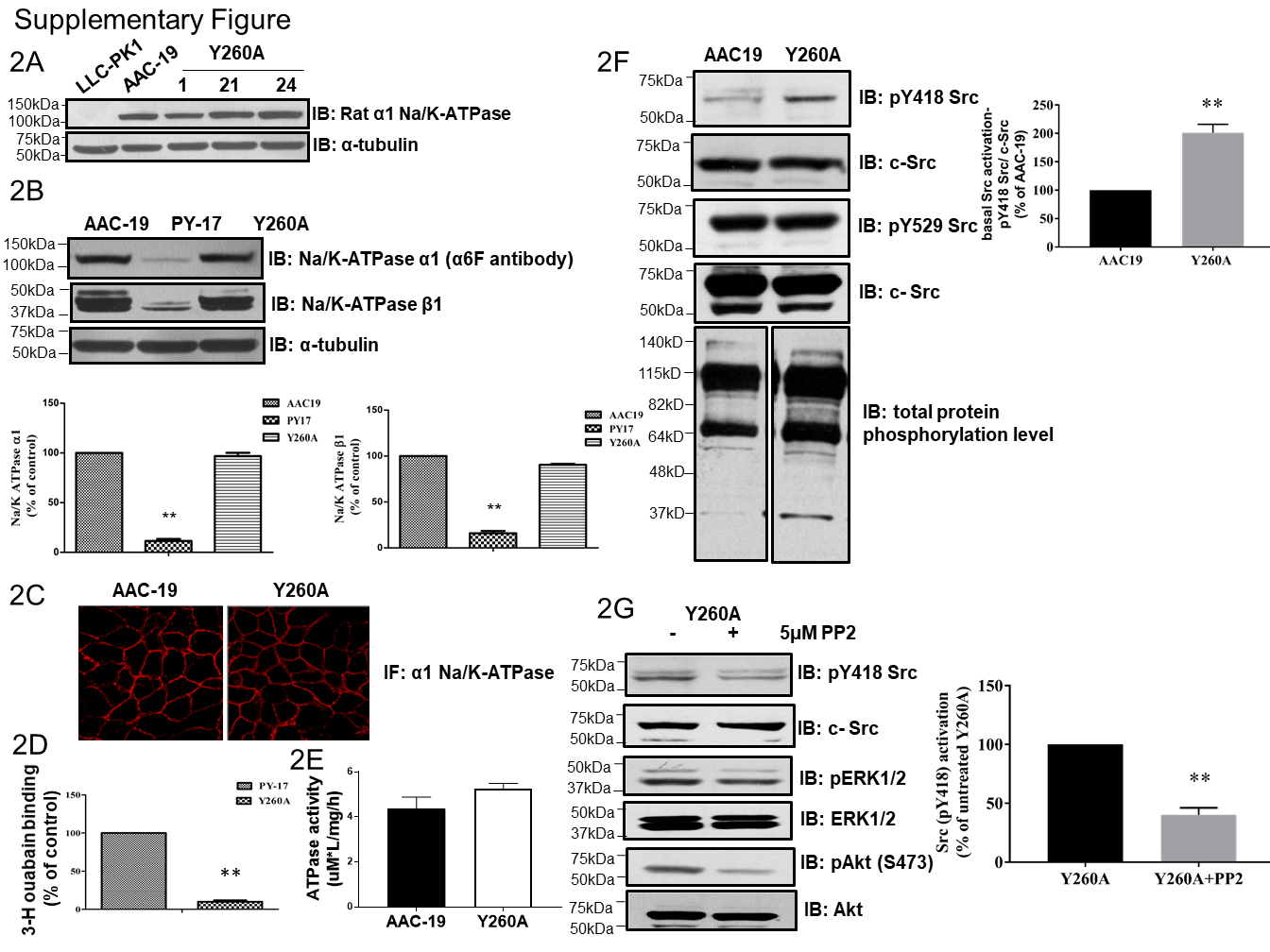
Supplementary Figure 2. (A) Expression of rat α1 Na/K-ATPase in different cell lines. Y260A mutant cell lines were generated as described in the “Methods”. Cell lysates from 3 different clones were analyzed for rat α1 Na/K-ATPase using a rat α1-specific antibody (NASE). Porcine LLC-PK1 and rat α1-rescued AAC-19 cells were used as a negative and positive control. Tubulin was probed as a loading control on the same membrane. Representative blots are shown, n=3. (B) α1 and β1 subunit expression in Y260A. Mutant clone 21 was compared with control AAC-19 and α1 knockdown cell line PY-17 using alpha6F antibody that recognizes both porcine and rat α1. **p<0.01 compared with AAC-19 (One-way ANOVA), n=3-4. (C) Immunofluorescence staining showing α1 Na/K-ATPase expression in the plasma membrane of AAC-19 and Y260A mutant clone 21 cells. (D) ^3^H ouabain binding study showing endogenous (porcine) α1 Na/K-ATPase expression in Y260A mutant clone 21 as compared with the parental α1 knockdown cell line PY-17. **p<0.01 as compared with PY-17 (Students T test), n=3. (E) Na/K-ATPase activity assay was performed as described in Methods, n=3. (F) Comparison of protein phosphorylation between AAC-19 and Y260A cells. Western blot showing Y418 and Y529 phosphorylation of Src kinase. Total Src was also probed as loading control. Quantitative data is shown on right, **p<0.01 to AAC-19 cells (Student T-test). n=6. Total protein tyrosine phosphorylation blots are shown at the bottom (Images are from different parts of the same gel- the full-length gel is shown in Supplementary Figure 7). (G) Protein phosphorylation in response to Src inhibition by PP2. Representative blots and quantification of Src activity (pY418) are shown. **p<0.01 to control (Student T-test).


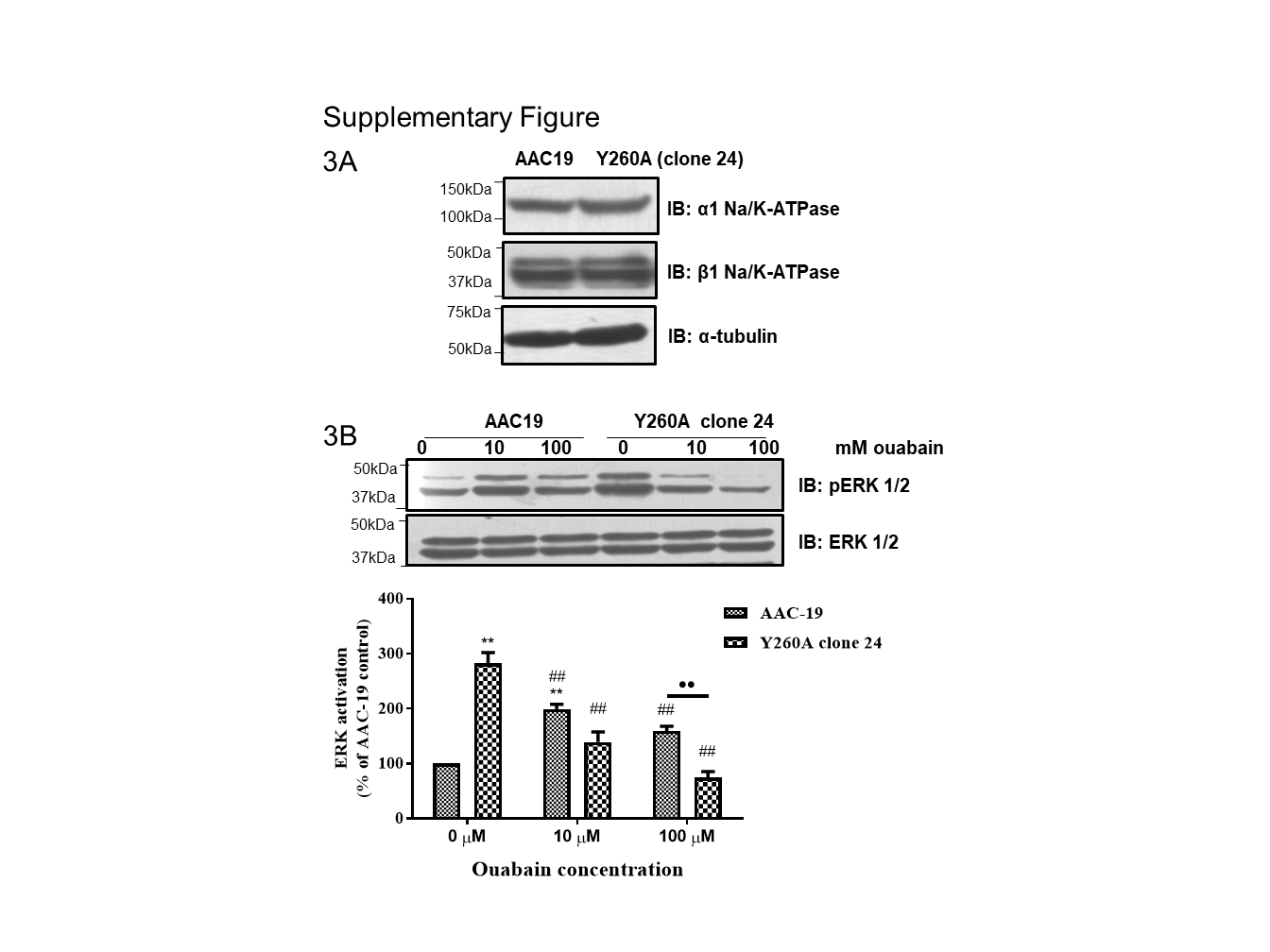


Supplementary Figure 3. (A) Total α1 and β1 subunit expression in Y260 mutant clone 24 as compared with control AAC-19. Representative blots are shown, n=3. (B) Western blot showing ouabain-induced ERK activation (pERK1/2/ERK 1/2) in AAC-19 and Y260A clone 24 cells. **p<0.01 compared with vehicle-treated control (the same cell line). ##p<0.01 compared with different cell lines, ••p<0.01 between different cell lines in the same treatment dose as indicated (Two-way ANOVA). n=3.


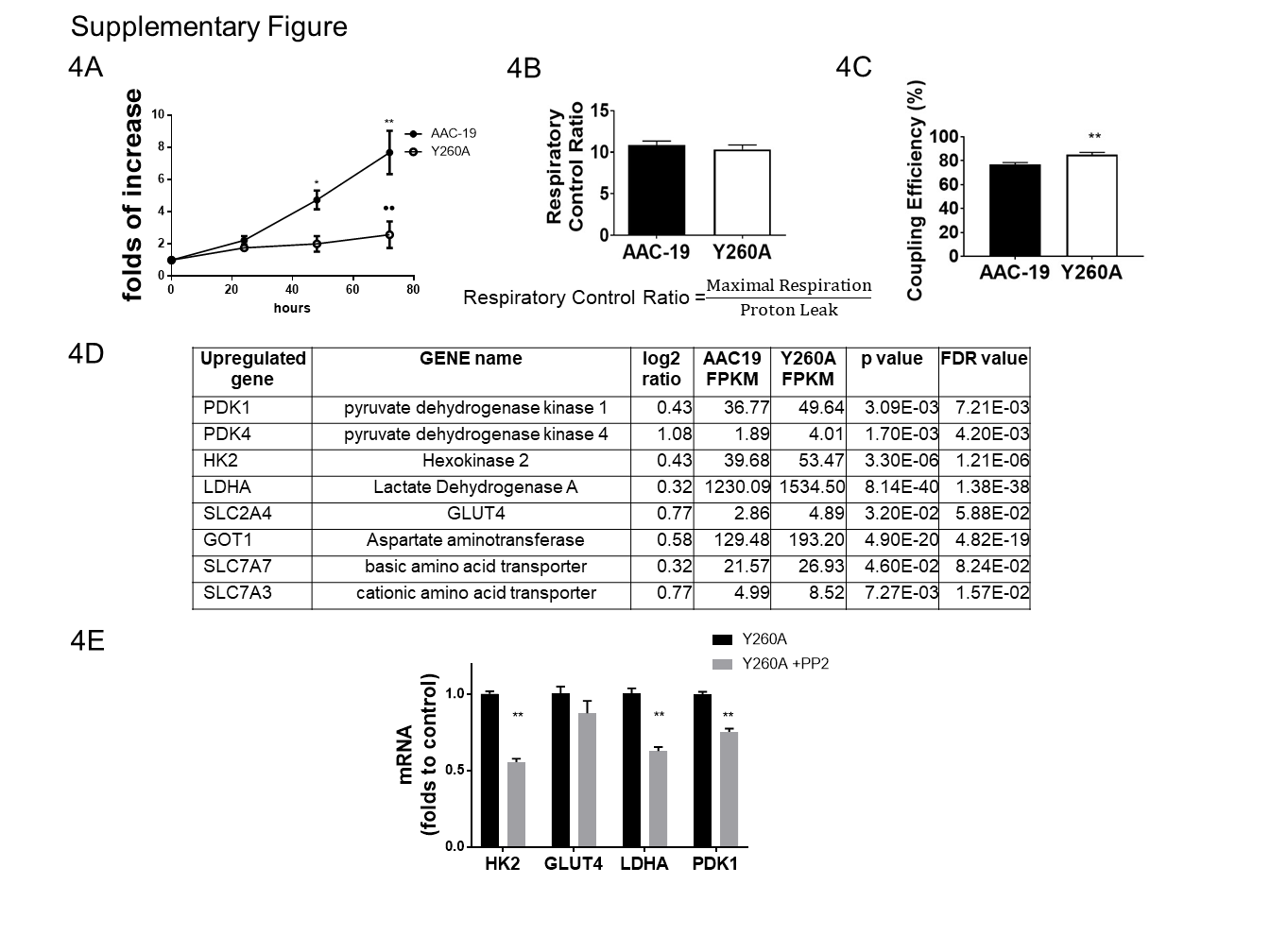
Supplementary Figure 4. (A) Comparison of cell proliferation rate of AAC-19 and Y260A cells. *p<0.05 and **p<0.01 compared to 0-hour in same cell line and ••p<0.01 compared between two cell lines at the same time point (Two-way ANOVA) n=4-5. (B) OCAR measurement of AAC-19 and Y260A. Maximal Respiration and Proton Leak data were generated and calculated from the OCAR measurements as described in “Methods”. Respiratory Control Ratio is calculated based on the equation as shown. (C) Coupling Efficiency as calculated from the OCAR measurements (right). **p<0.01 compared with control AAC-19. n=3-4. (D) RNAseq analyses showing upregulated genes involved in metabolic switch in Y260A mutant cells, as compared with AAC-19. Upregulation is expressed as Log2 ratio. (E) Y260A cells were treated with 5μM PP2 for overnight and qPCR was used to measure mRNA expression level of different genes. ** p<0.01 compared with untreated control, n=3.


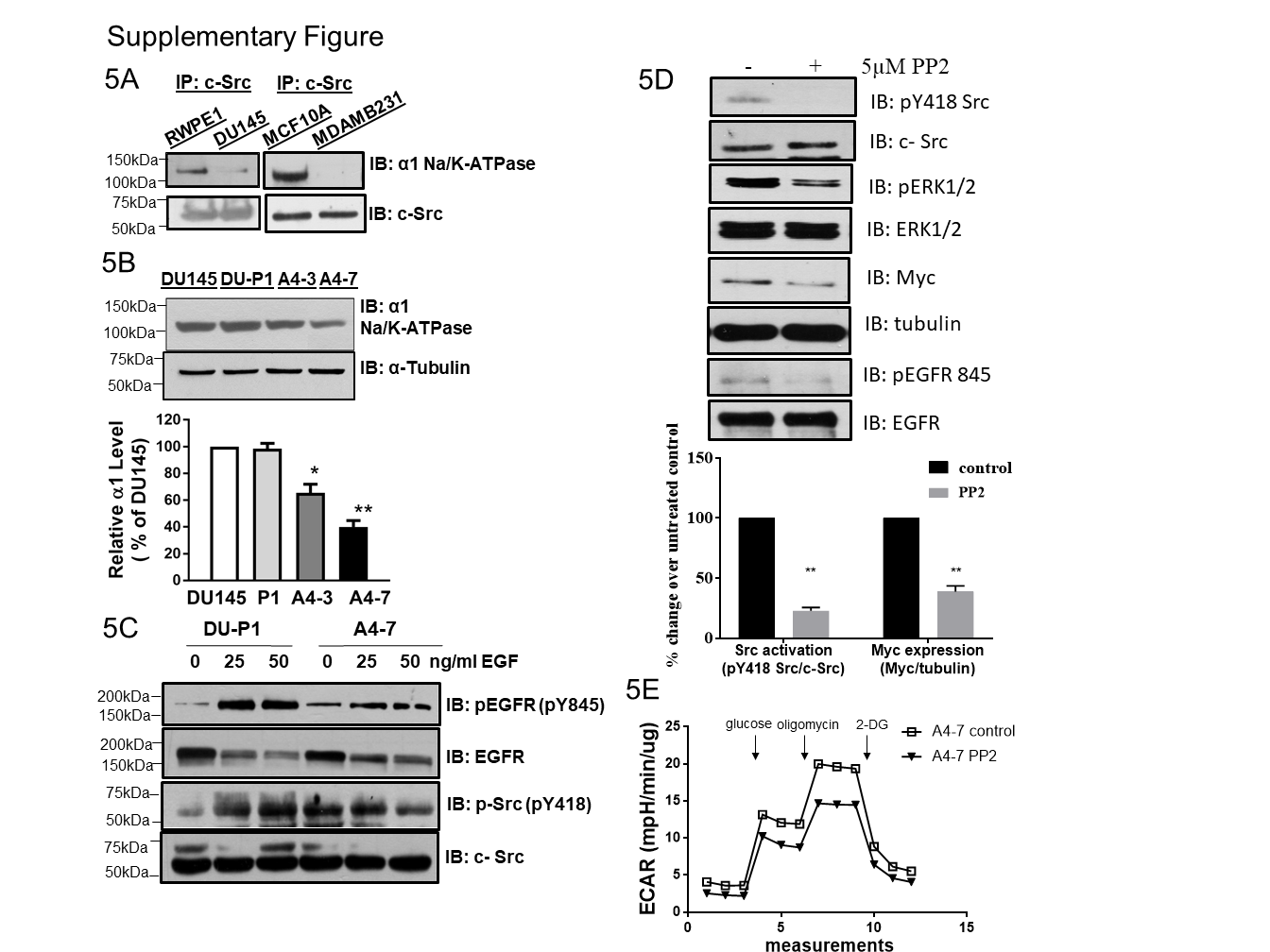


Supplementary Figure 5 (A) Coimmunoprecipitation to compare α1 Na/K-ATPase/Src kinase interaction in normal prostate RWPE1 vs. prostate cancer cell line DU145 (on left) and in normal breast MCF10A vs. breast cancer cell line MDAMB231 (on right). Representative blots are shown, n=3. (B) Western blot showing α1 Na/K-ATPase expression in DU145, DU-P1 (control) and α1- knockdown cell lines (A4-3 and A4-7). (C) EGF stimulation of DU-P1 and A4-7 cells. Cells were exposed to different concentrations of EGF for 5 minutes and Src activation (pY418 Src/c-Src) and EGFR activation at Src-mediated phosphorylation site (pY845 EGFR/EGFR) were measured by Western blot. Representative blots are shown, n=3-4. (D) A4-7 cells were treated with 5μM PP2 for 90m and lysates from control and treated cells were assayed for the activation of Src, ERK and EGFR. The same lysates were also probed for Myc expression. Quantitative data are shown on right. **p<0.01 compared with untreated control, n=3. (E) ECAR measurement of A4-7 cells cultured in the presence or absence of PP2 (5μM, 4h).


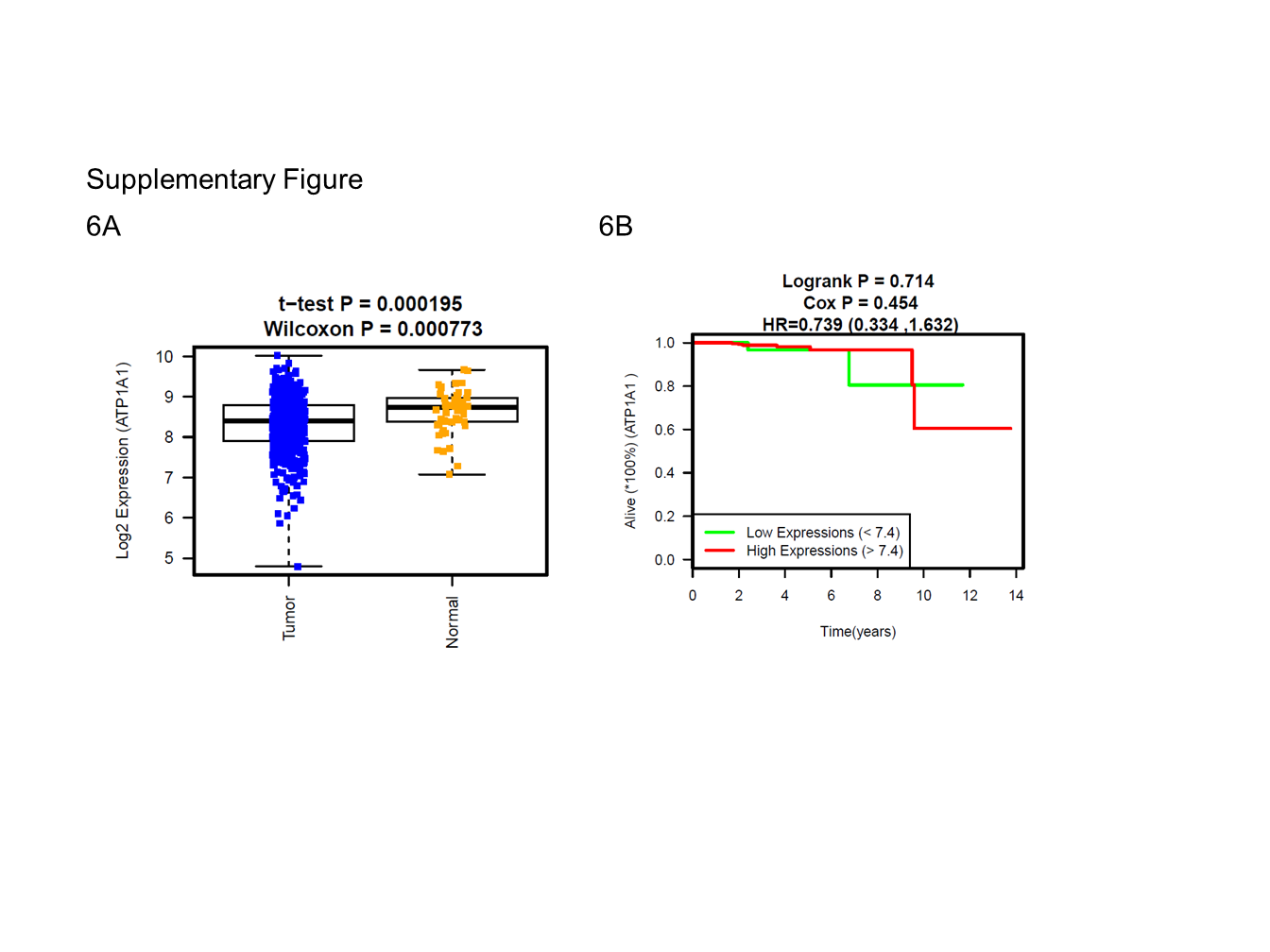


Supplementary Figure 6. (A) Na/K-ATPase α1 (ATP1A1) gene expression was decreased in prostate cancer compared with normal prostate in the TCGA-PRAD database. n=495. (B) A decrease in α1 gene expression in the same database based on best cutoff value was not correlated with patient survival.


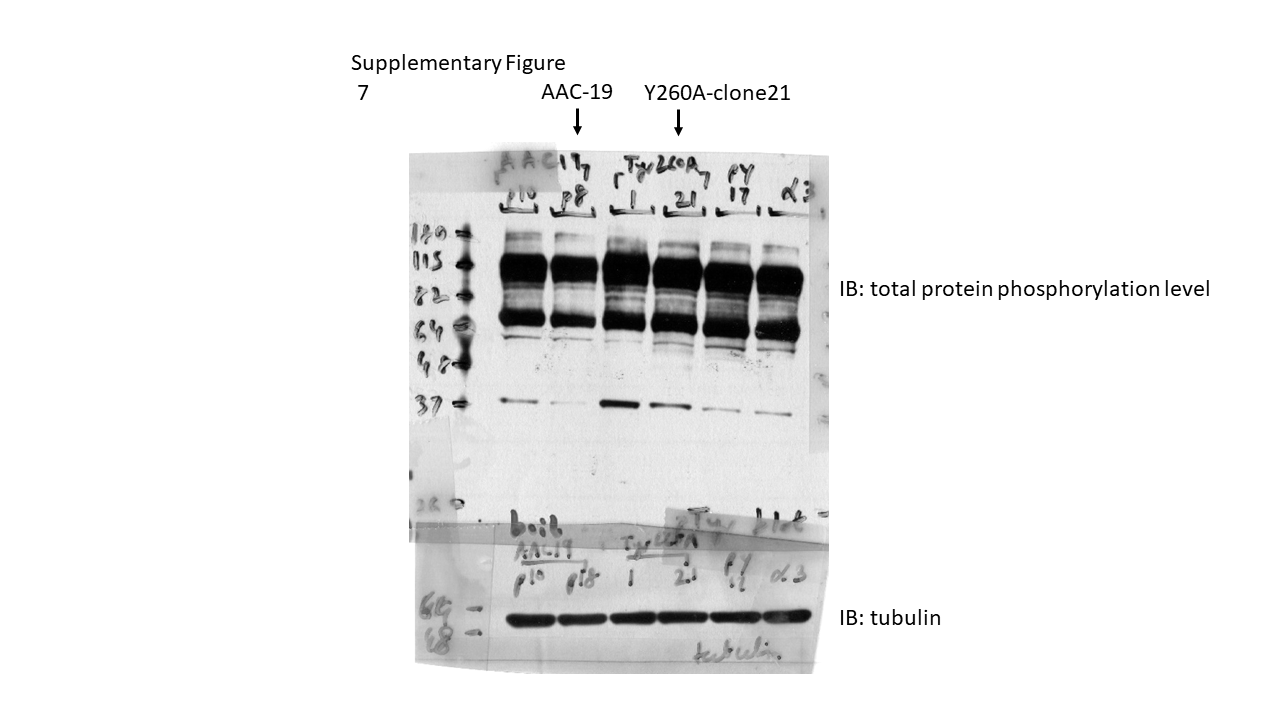


Supplementary Figure 7. Full- length blot of the total protein phosphorylation level in Supplementary Figure 2F.
